# Gallbladder agenesis with hepatic impairment: a case report

**DOI:** 10.1186/s12887-018-1343-0

**Published:** 2018-11-19

**Authors:** Yoshihiko Takano, Mirei Hoshino, Sakae Iriyama, Kyoko Takayanagi, Manabu Ishiro, Nobuhiro Kawakami, Takayuki Okamura

**Affiliations:** Department of Pediatrics, Sakai City Medical Center, 1-1-1 Ebaraji-Cho, Nishi-Ku, Sakai City, Osaka, 593-8304 Japan

**Keywords:** Gallbladder agenesis, Hepatic impairment, Imaging modalities, Mycoplasma pneumoniae

## Abstract

**Background:**

Gallbladder agenesis is a rare congenital malformation. More than 50% of cases are isolated and asymptomatic. These asymptomatic patients are principally healthy and need no interventions. However, some patients develop symptoms, presenting with clinical signs and complaints similar to those of biliary tract disease. Symptoms commonly occur in the fourth or fifth decade of life of the patient. At the present time, gallbladder agenesis is diagnosed using a combination of imaging modalities, without surgical intervention, to avert serious complications following surgery.

**Case presentation:**

We describe a 13-year-old Japanese girl with a history of recurrent hepatic impairment, which had not been thoroughly investigated. She was referred to our hospital following 2 days of fever, fatigue, and abnormal blood tests suggested impaired liver function. Data from chest X-ray findings combined with a positive loop-mediated isothermal amplification assay result indicated *Mycoplasma pneumoniae* pneumonia, which was treated with oral azithromycin. To investigate potential hepatic impairment, we performed several imaging studies, namely, abdominal ultrasonography, magnetic resonance cholangiopancreatography, and contrast enhanced computed tomography. These imaging studies revealed a normal liver; however, the gallbladder was not in the usual nor any aberrant position in imaging investigations of the patient. Based on these results, we diagnosed gallbladder agenesis; however, the etiology of her hepatic impairment has not been elucidated.

**Conclusion:**

We present a case of gallbladder agenesis with hepatic impairment, where the diagnosis was made without surgical intervention. Clinicians should perform a detailed investigation when they encounter repeated hepatic impairment.

## Background

Gallbladder agenesis (GA) is a rare congenital anomaly that is caused by failure of the cystic bud to develop in the embryonic period. Clinically, GA is classified into three types: (1) multiple fetal anomalies causing perinatal mortality, (2) symptomatic, and (3) asymptomatic [1]. Symptomatic patients usually present with complaints similar to those of patients with biliary tract diseases, namely, right upper quadrant abdominal pain, jaundice, nausea, vomiting, fatty food intolerance, or dyspepsia. Although the prevalence of each of the three types varies according to the literature, approximately 70% of GA cases are asymptomatic isolated anomalies [2, 3]. Most patients with asymptomatic GA are healthy and do not experience hepatic impairment; therefore, they need no intervention. However, some of them become symptomatic and require surgical intervention at some point during the course of their life. This occurs most commonly in the fourth or fifth decade [4]. Diagnosis of GA in such a population is often incidental during a surgical procedure on the abdomen or during autopsy.

Previously, GA was diagnosed only at laparotomy, after excluding ectopic gallbladder and performing intraoperative cholangiogram. However, in 2010, Malde proposed an algorithm for the investigation of suspected GA to promote preoperative diagnosis to prevent unnecessary surgical procedures that carry a risk of iatrogenic injury [5]. Therefore, GA is currently diagnosed less invasively using a combination of laparoscopy, endoscopic ultrasound, computed tomography, and magnetic resonance cholangiopancreatography (MRCP). Kasi et al. and Tagliaferri et al. reported GA cases wherein the diagnosis was made using only diagnostic imaging techniques without laparoscopic survey [2, 6]. With the advent of newer imaging modalities, more GA cases are diagnosed before surgical interventions.

Here, we present a case of asymptomatic GA diagnosed using only imaging modalities, while conducting examinations for hepatic impairment comorbid with *Mycoplasma pneumoniae* pneumonia.

## Case presentation

A 13-year-old Japanese girl was admitted to our hospital due to fever, fatigue and abnormal blood test results. She had a history of chronic constipation and repeated (once or twice a year) intermittent hepatic impairment associated with acute febrile illnesses, but she had not received any detailed examination for these symptoms. Her latest medical record showed that she presented to her family doctor 15 months prior to admission to our hospital, following a febrile episode. Her laboratory results for aspartate transaminase (AST), alanine transaminase (ALT), total bilirubin (T-Bil), and alkaline phosphatase (ALP) showed levels of 406 IU/L (reference range 14–29 IU/L), 227 IU/L (reference range 9–28 IU/L), 0.5 mg/dL (reference range 0.25–1.20 mg/dL), and 798 IU/L (reference range 220–1250 IU/L), respectively. Her hepatic impairment resolved spontaneously after the fever subsided. Other medical and family histories were unremarkable. The day before her admission, she had visited her family doctor complaining of fever for 2 days and weariness. On the following day she remained febrile and vomited three times; following this, she consulted the emergency pediatric service at night, where blood analysis was performed. It revealed abnormal liver function test results, with AST levels of 681 IU/L and ALT levels of 547 IU/L; therefore, she was referred to our hospital for a thorough investigation. On admission, she had an axillary temperature of 37.9 °C and tachypnea of 30 per minute. Other physical examination findings were normal. Although she had no apparent respiratory symptoms other than tachypnea, we performed a chest X-ray and mycoplasma loop-mediated isothermal amplification (LAMP) assay because *Mycoplasma pneumoniae* pneumonia was locally prevalent. Chest X-ray revealed slight bilateral reticular opacity, which is a clinical feature suggestive of viral or atypical pneumonia.

Following a positive result in the LAMP assay, we diagnosed her with *Mycoplasma pneumoniae* pneumonia, and oral azithromycin was initiated. The fever and weariness of the patient completely subsided with medication and resting; she was discharged after 4 days of admission.

To elucidate the etiology of her hepatic impairment, we performed blood analysis on admission, which revealed AST levels of 394 IU/L, ALT levels of 401 IU/L, T-Bil levels of 0.77 mg/dL, ALP levels of 885 IU/L, and gamma glutamyl transferase (γ-GTP) levels of 146 IU/L (reference range 8–36 IU/L) (Table 1).

**Table 1:** Hepatobiliary test results of blood examination

	Age-related reference range	On admission	4 days	2 weeks	6 weeks
AST	14–29 (IU/L)	394	46	28	29
ALT	9–28 (IU/L)	401	101	21	9
ALP	220–1250 (IU/L)	885	527	532	415
γ-GTP	8–36 (IU/L)	146	81	47	13
T-Bil	0.25–1.20 (mg/dL)	0.77	0.29	0.44	0.90

Complete blood cell counts, coagulation test results, antinuclear antibody titer, and viral tests for hepatitis viruses, Epstein-Barr virus and cytomegalovirus were all normal. Serum levels of copper, ceruloplasmin, and alpha-fetoprotein are unremarkable.

**Fig. 1:**
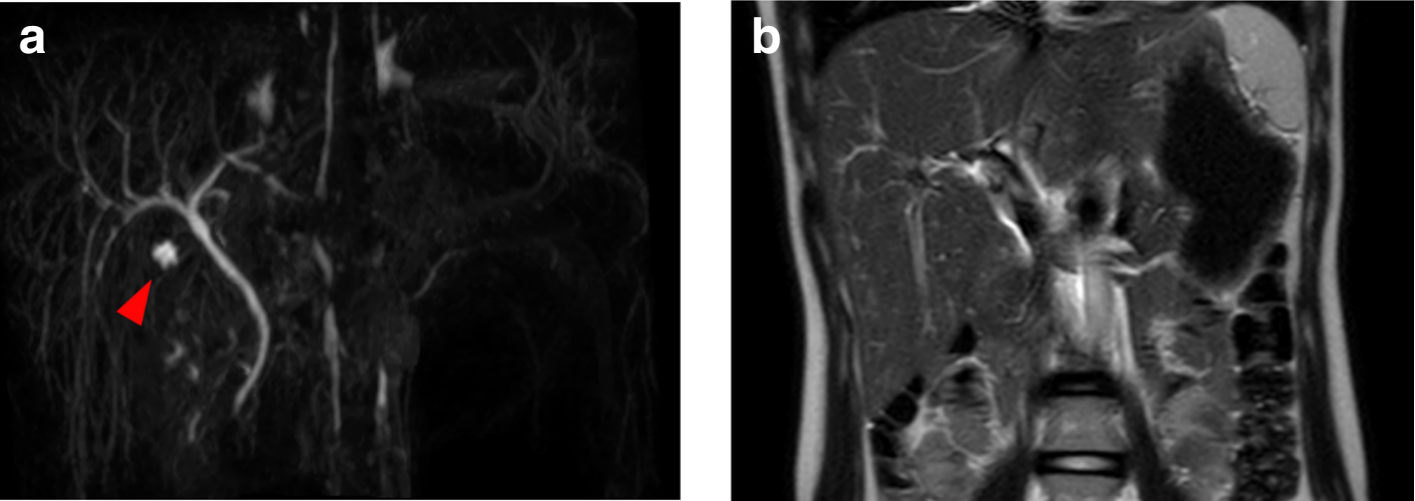
Magnetic resonance cholangiopancreatography (MRCP) carried out on the fourth day of admission. The MRCP images could depict neither the gallbladder nor the cystic duct. The study showed no pancreaticobiliary maljunction. A hepatic cyst, which is a normal variant, was detected (shown by the arrow head). The cyst showed no continuity with the common bile duct. **a** Coronal image at maximum intensity projection, **b** T2 weighted coronal image

With respect to imaging studies, initial abdominal ultrasonography revealed a normal liver; however, the gallbladder could not be visualized. A second abdominal ultrasonography was performed following fasting; however, visualization of the gallbladder was impossible. MRCP was carried out on the fourth day of admission, which revealed no major abnormality except for the absence of the gallbladder (Fig. 1).

**Fig. 2:**
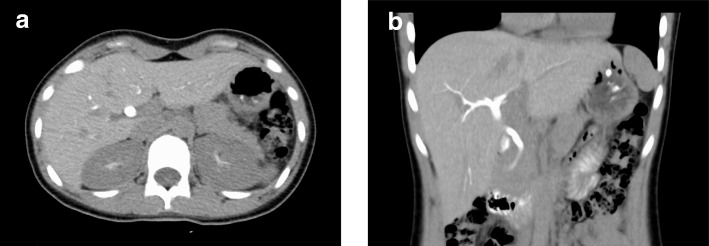
Drip infusion cholecystocholangiography with computed tomography (DIC–CT) performed at 6 weeks after discharge. The bile ducts were analyzed by DIC-CT using a radiopaque dye as a contrast medium. We intravenously administered the dye, which accumulates preferentially in the tissue of gallbladder or bile ducts. The dye did not show any silhouette of the gallbladder, indicating the absence of the gallbladder. **a** Axial view, **b** coronal view

We performed further studies on an outpatient basis, which revealed no anomaly other than the absence of the gallbladder. Six weeks after discharge, her laboratory tests showed normal levels of AST, 29 IU/L; ALT, 9 IU/L; T-Bil, 0.90 mg/dL; ALP, 415 IU/L; and γ-GTP, 13 IU/L (Table 1). To confirm the absence of the gallbladder, we performed drip infusion cholecystocholangiography with computed tomography (DIC–CT); this imaging study uses a radiopaque dye that preferentially accumulates in the tissue of the gallbladder or bile ducts. The DIC-CT study could not reveal the gallbladder in the usual nor aberrant position (Fig. 2).

Based on these results, the patient was diagnosed with gallbladder agenesis. During outpatient follow-up, she gradually manifests nausea after eating fatty foods; therefore, we prescribed smooth muscle relaxant and ursodeoxycholic acid. The outpatient follow-up is still ongoing, and febrile episodes have not been observed after discharge.

## Discussion

We present a case of GA where the diagnosis was made thanks to hepatic impairment found at emergency service, although the patient showed no apparent biliary symptoms. Despite experiencing repeated episodes of hepatic impairment, she had never been investigated thoroughly. Our attempt to elucidate the etiology of hepatic impairment led to an incidental diagnosis of GA. Clinicians should seek detailed investigation for potential underlying diseases when they encounter repeated episodes of hepatic impairment.

We diagnosed GA using only imaging modalities, without performing laparoscopy or laparotomy. The imaging studies performed in our case revealed a normal liver image; however, they revealed the absence of the gallbladder in its usual position, and the possibility of ectopic gallbladder was excluded based on the findings of MRCP [7]. In addition, the possibility of contracted gallbladder due to chronic cholecystitis was disregarded because the patient did not have any history of recurrent abdominal pain. Although the diagnosis may be inconclusive, laparoscopy should not be performed in patients with no severe symptoms solely to obtain a more robust diagnosis, since laparoscopy is an invasive procedure. Hence, we concluded that laparoscopy is not indicated at this stage and diagnosed the patient with GA, similar to the diagnosis made by Kasi et al. and Tagliaferri et al. [2, 6].

Although we cannot clearly explain why the febrile illnesses of our patient repeatedly induced hepatic impairment, we believe that this finding may be relevant to GA. Generally, GA is not associated with hepatic impairment as in cases post cholecystectomy; however, patients with GA frequently develop biliary dyskinesia or choledocholithiasis [3–5]. One possible etiology of the hepatic impairment in our case is the presence of cholestasis due to biliary dyskinesia or undiagnosed common bile duct stones, which could lead to transient hepatic impairment. Although no apparent bile duct stones were detected in our patient, the sensitivity of both MRCP and DIC-CT is not adequate for the detection of choledocholithiasis. Zidi et al. reported that the sensitivity of MRCP for the detection of bile duct stones smaller than 6 mm is as low as 33.3% [8]; therefore, our patient might have undiagnosed bile duct stones. In our case, the diameter of the common bile duct in abdominal ultrasonography at 4 months after discharge was 4.4 mm; the common bile duct was thought to be slightly dilated since the diameter was higher than the age-adjusted reference range, which is from 1.5 mm to 4.1 mm [9]. The gradual manifestation of fatty food intolerance may be associated with potential choledocholithiasis; however, the symptoms were not too severe, and consent for endoscopic retrograde cholangiopancreatography was not obtained at the time of writing this manuscript. Therefore, the patient is being closely followed-up along with a prescription of smooth muscle relaxant that can decrease tonus of the sphincter of Oddi and ursodeoxycholic acid that can help dissolve bile duct stones. Hereafter, we will continue outpatient follow-up at appropriate intervals and determine if invasive procedures are necessary should the patient manifest more severe symptoms.

In our case, hepatic impairment associated with *Mycoplasma pneumoniae* pneumonia served as a clue in diagnosing GA. There is, to our knowledge, no medical literature that described comorbidity of GA and *Mycoplasma pneumoniae* pneumonia. We diagnosed the patient with *Mycoplasma pneumoniae* pneumonia following a positive result in the LAMP assay and confirmed the elevation of Mycoplasma antibody titers (data not shown); hence, we are confident that the fever of our patient was due to *Mycoplasma pneumoniae* pneumonia. Taking into consideration her past medical history of repeated hepatic impairment occurring once or twice a year, we think that her hepatic impairment derives nonspecifically from febrile illnesses. Therefore, we do not think that her hepatic impairment was directly caused by *Mycoplasma pneumoniae* pneumonia, although *Mycoplasma pneumoniae* infection is sometimes followed by hepatic impairment as an extrapulmonary manifestation.

## Conclusion

We present a case of GA with recurrent hepatic impairment, wherein the diagnosis was made without surgical intervention. Clinicians should make every effort to detect potential underlying diseases when they encounter a case of repeated hepatic impairment.

## Abbreviations

ALP: Alkaline phosphatase
ALT: Alanine transaminase
AST: Aspartate transaminase
DIC-CT: Drip infusion cholecystocholangiography with computed tomography
GA: Gallbladder agenesis
LAMP: Loop-mediated isothermal amplification
MRCP: Magnetic resonance cholangiopancreatography
T-Bil: Total bilirubin
γ-GTP: Gamma glutamyl transferase
